# Potato StLecRK-IV.1 negatively regulates late blight resistance by affecting the stability of a positive regulator StTET8

**DOI:** 10.1093/hr/uhac010

**Published:** 2022-02-11

**Authors:** Lei Guo, Yetong Qi, Yang Mu, Jing Zhou, Wenhe Lu, Zhendong Tian

**Affiliations:** 1 College of Agronomy, Northeast Agricultural University, Harbin, 150030, China; 2 Key Laboratory of Horticultural Plant Biology (HZAU), Ministry of Education, Huazhong Agricultural University (HZAU),Wuhan, 430070, China; 3 Key Laboratory of Potato Biology and Biotechnology (HZAU), Ministry of Agriculture and Rural Affairs, Huazhong Agricultural University,Wuhan, 430070, China; 4 Potato Engineering and Technology Research Center of Hubei Province, Huazhong Agricultural University, Wuhan, 430070, China; 5 Hubei Hongshan Laboratory. Huazhong Agricultural University, Wuhan, 430070, China

## Abstract

Plant receptor-like kinases (RLKs) regulate many processes in plants. Many RLKs perform significant roles in plant immunity. Lectin receptor-like kinases (LecRLKs) are a large family of RLKs. However, the function of most LecRLKs is poorly understood. In this study, we show that a potato LecRLK, StLecRK-IV.1, is involved in plant immunity against *Phytophthora infestans*. As a negative regulator of immunity, *StLecRK-IV.1* is downregulated by *P. infestans* and activated by abscisic acid. The transient expression of StLecRK-IV.1 in *Nicotiana benthamiana* enhanced *P. infestans* leaf colonization significantly. In contrast, the size of disease lesions caused by *P. infestans* was reduced by virus-induced gene silencing of the *StLecRK-IV.1* ortholog in *N. benthamiana*, *NbLecRK-IV.1*, as well as in potato plants with stable RNA interference of *StLecRK-IV.1*. Tetraspanin-8 (StTET8) was identified to be interacting with StLecRK-IV.1 using a membrane yeast two-hybrid system, which was further verified by co-immunoprecipitation, a luciferase complementation assay, and a bimolecular fluorescence complementation test. StTET8 is a positive immune regulator that restrains *P. infestans* infection. The co-expression of StLecRK-IV.1 with StTET8 antagonized the positive roles of StTET8 against *P. infestans.* Moreover, the co-expression of StTET8 with StLecRK-IV.1 affected the stability of StTET8, which was confirmed by a western blot assay and confocal assay. Taken together, our work first reveal that a potato L-type lectin RLK, StLecRK-IV.1, negatively regulates plant immunity by targeting a positive regulator, StTET8, through affecting its stability.

## Introduction

The plant membrane-associated pattern recognition receptors (PRRs) play pivotal roles in regulating immune responses by perceiving pathogen-associated molecular patterns (PAMPs) or host-derived damage-associated molecular patterns (DAMPs) and ensuing activating or inhibiting downstream signal transduction to ward off microbes [1]. Plant PRRs include receptor-like kinases (RLKs) and receptor-like proteins (RLPs) [1]. RLKs consist of an extracellular domain, a transmembrane domain, and an intracellular kinase domain, but RLPs lack the latter. According to the characteristics of the ligands recognized, PRRs generally contain extracellular domains such as leucine-rich repeats (LRRs), lectin-like motifs, lysin motifs (LysMs), or epidermal growth factor (EGF)-like domains [1, 2].

Lectin receptor-like kinases (LecRKs) contain an extracellular Lectin_legB domain. The LecRK family of PRRs is quite extensive. In *Arabidopsis thaliana*, 75 LecRK members are grouped into three categories, including 1 C-type, 32 G-type, and 42 L-type LecRKs. Among them, the 42 L-type LecRKs are further classified into nine major clades [3, 4]. Plant LecRKs play vital roles in immunity throughout the plant kingdom [5]. In *Arabidopsis*, AtLecRK-IX.1 and AtLecRK-IX.2 were found to positively regulate resistance to *Phytophthora* [6, 7]. AtLecRK-VI.2 also contributes to disease resistance against the necrotrophic bacterium *Pectobacterium carotovorum* and the hemibiotrophic *Pseudomonas syringae* by activating the pattern-triggered immunity (PTI) response [8]. AtLecRK-I.9 regulates jasmonic acid signaling components and perception of ATP in the face of invasion by *P. syringae* [9, 10]. As a potential host target of an RXLR effector, overexpression of* AtLecRK-I.9* enhances resistance to both *Phytophthora brassicae* and *Phytophthora infestans* [11, 12]. AtLecRK-V.2 and AtLecRK-VII.1 enhance the resistance of plants via a function in stomatal immunity [13]. Overexpressing *AtLPK1 (AtLecRK-IV.3)* enhances resistance to *Botrytis cinerea* infection and results in increased expression of a collection of defense-related genes [14]. AtLecRK-V.5 adversely affects closure of stomata on bacterial infection, and overexpression of LecRK-V.5 in *Arabidopsis* leads to enhanced susceptibility to *P. syringae* pv. *tomato* DC3000 [15]. Some *Arabidopsis* LecRLKs have been reported to contribute to *P. infestans* resistance in solanaceous plants [11, 16]. Besides these, there are also a few LecRLKs studied in Solanaceae plants. In *Nicotiana benthamiana*, NbLRK1 was activated during defense responses and interacted with *P. infestans* elicitin INF1 and inhibits the hypersensitive response induced by INF1 [17, 18]. Clade IX LecRLKs in tomato and *N. benthamiana* are involved in *Phytophthora* resistance [19]. A pepper CaLecRK-S.5 has been found to confer broad-spectrum resistance by initiating activation and positively functioning in the defense response mediated by *Phytophthora* elicitin [20].

Tetraspanins, belonging to the transmembrane 4 superfamily (TM4SF), as integral membrane components for endosome organization, are widely distributed in mammals, insects, fungi, mosses, and higher plants [21]. As transmembrane junction proteins, tetraspanins are involved in intercellular and cell-to-cell signal transduction and participate in transport processes, membrane fusion, membrane recognition, and mutualistic communication in host plants and animals [22–24]. As the indispensable component of the extracellular vesicles (EVs), tetraspanins are considered to be key players in the intercellular communication of transferring proteins, RNAs, and lipids. These intercellular communications are involved in biological and non-biological stress responses, particularly in plant immune responses [25]. In mammals, tetraspanins CD63, CD81, and CD9 were considered as specific exosome markers [26]. There are 17 *Tetraspanin (TET)*-like genes encoded in the *Arabidopsis* genome [21], of which *TET8* and *TET9* are mammalian CD63 orthologs. The *Arabidopsis tet8* mutant shows the reduced formation of EVs accompanied by impaired reactive oxygen species (ROS) burst in response to stressors, suggesting the role of TET8 in EV creation [27]. Like CD63 in mammals, TET8 is considered a specific marker for exosomes in plants, and TET8-associated EVs can be considered plant exosomes. *B. cinerea* induces TET8 and TET9-associated vesicle accumulation at the sites of infection. TET8- and TET9-associated exosomes contribute to plant immunity against *B. cinerea* infection by transferring host small RNAs (sRNAs) into fungal cells to suppress pathogenicity by targeting virulence genes [28].

Potato late blight is a severe danger to potato production and global food security, caused by the destructive *P. infestans* [29]. Many reports revealed that LecRLKs play very important roles in plant–pathogen interactions against diverse pathogens [4]. In potato, 113 *LecRLKs* were identified, including 2 C-type, 26 L-type, and 85 G-type members [30]. However, their function, especially in plant immunity, has been poorly characterized. As a novel resource of cell surface receptors, LecRLKs have the potential to be utilized to improve the durability of potato resistance against *P. infestans* [5]. In our previous work, we found that transient expression of *StLecRK-IV.1* in *N. benthamiana* leaves significantly promotes the colonization of *P. infestans*. Here we further study the functional mechanism of StLecRK-IV.1 in the regulation of plant immunity. We demonstrate that a potato L-type lectin RK, StLecRK-IV.1, negatively regulates plant immunity by affecting the stability of one of its interacting proteins, StTET8, a positive regulator of immunity to *P. infestans*.

## Results

### Structure, localization, and induction pattern of StLecRK-IV.1

**Figure 1:**
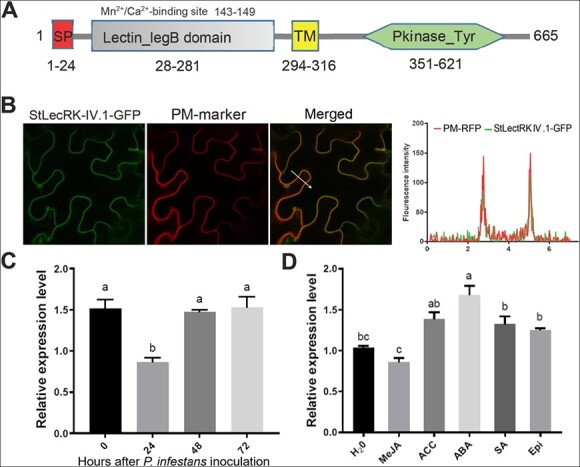
StLecRK-IV.1 structure, localization, and induction pattern. **a** Schematic structure of StLecRK-IV.1. SP, signal peptide; TM, transmembrane domain, Pkinase_Tyr, Tyr kinase. **b** StLecRK-IV.1 localizes on the plasma membrane (PM). Left to right: green channel (StLecRK-IV.1-GFP), orange channel (OFP-CBL1n, PM-marker), merge channel, StLecRK-IV.1 (green), and OFP-CBL1n (orange) fluorescence intensity plots across white arrow. StLecRK-IV.1-GFP co-localized with OFP-CBL1n. *N. benthamiana* leaves were agroinfiltrated by GV3101 containing StLecRK-IV.1-GFP and CBL1n constructs. **c** Relative expression levels of StLecRK-IV.1 in response to *P. infestans*. Leaves were sampled at 0, 24, 48, and 72 hours after *P. infestans* inoculation. **d** Relative expression levels of *StLecRK-IV.1* in potato in response to plant defense hormones. Leaves were collected 6 hours after treatments with 1 mM ABA, 0.05 mM brassinolides [applied as epibrassinolide (Epi)], 1 mM ethylene (ET, applied as ACC), 1 mM salicylic acid (SA), 1 mM methyl jasmonate (MeJA), and ddH_2_O. All solutions contained 1% DMSO. The samples were used to extract RNA. qRT–PCR was used to detect transcript accumulations. The combined data are from three biological repeats. In **c** and **d**, error bars indicate mean ± standard error of the mean (*n* = 3). Lower-case letters above the bars represent significant differences (one-way ANOVA and Tukey’s HSD test: *P* < .05).

*StLecRK-IV.1* was cloned from potato material DM1-3. This gene encodes a protein that shares 60% of its identity with AtLecRK-IV.1 (*Arabidopsis* L-type lectin domain containing receptor kinase IV.1, NP_181307.1) and thus was named *StLecRK-IV.1* (XP_006341207.2). Structural prediction showed that StLecRK-IV.1 contains a typical LecRLK structure, including a signal peptide, an N-terminal extracellular Lectin_legB domain, a transmembrane domain, and a predicted tyrosine kinase domain (Fig. 1a). The Lectin_legB domain has saccharide binding sites and a hydrophobic cavity structure, suggesting that it may be involved in the interaction with hydrophobic ligands. It also contains potential Ca^2+^ and Mn^2+^ binding sites (at amino acid positions 143–149), which can stabilize the saccharide binding sites (Fig. 1a).

Phylogenetic analysis was performed to investigate the relationship between StLecRK-IV.1 and the LecRK family members from *N. benthamiana*, *Solanum lycopersicum*, and *Arabidopsis.* StLecRK-IV.1 was grouped in clade IV with four *Arabidopsis* LecRLKs and other orthologs from *N. benthamiana* and *S. lycopersicum*. In particular, StLecRK-IV.1 shares 72% of its identity with a *N. benthamiana* lectin receptor-like kinase (NbS00015931g0001.1) in this clade (Supplementary Data Fig. 1).

LecRKs belong to the receptor-like kinases. To investigate the cellular localization of StLecRK-IV.1, StLecRK-IV.1-GFP, made by fusing green fluorescent protein (GFP) to the C-terminal of StLecRK-IV.1, was co-overexpressed in *N. benthamiana* together with a plasma membrane marker, OFP-CBL1n [31]. StLecRK-IV.1-GFP was observed by confocal microscopy to co-localize with OFP-CBL1n in the plasma membrane (Fig. 1b).

To investigate the response of *StLecRK-IV.1* to *P. infestans*, leaves of potato variety ‘E-potato-3’ (E3) were inoculated with *P. infestans* isolate HB09-14-2. *StLecRK-IV.1* was downregulated at 24 hours post-inoculation and then restored to normal level (Fig. 1c). Hormones are vital substances for regulating plant innate immunity [32, 33]. To test whether the expression level of *StLecRK-IV.1* is regulated by hormones, potato E3 leaves were treated with various plant defense hormones, and qRT–PCR was performed to measure the expression level of *StLecRK-IV.1*. The results show that *StLecRK-IV.1* was upregulated by abscisic acid (ABA) and amino cyclopropanecarboxylic acid (ACC, a precursor in ethylene biosynthesis) to a lesser extent (Fig. 1d), suggesting a possible role of LecRK-IV.1 linking to the ABA and ethylene signaling pathways.

### StLecRK-IV.1 negatively regulates plant resistance against *P. infestans*

**Figure 2:**
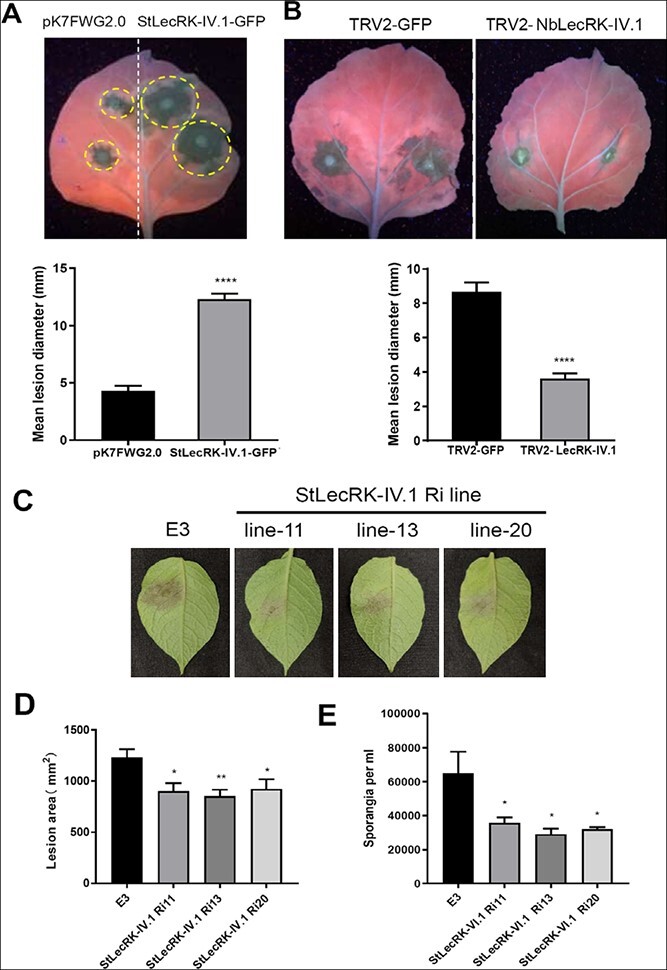
StLecRK-IV.1 promotes *P. infestans* colonization. **a** Graph presenting transient overexpression of StLecRK-IV.1-GFP markedly suppressed the disease resistance of *N. benthamiana* against *P. infestans* compared with the control (empty vector). Demonstrative images (under UV light) were taken at 7 dpi following *P. infestans* isolate 88069 inoculation. Mean lesion diameter (mm) at sites of transient expression of StLecRK-IV.1-GFP and control was measured at 5 dpi. Values are means ± standard deviation (two-tailed t-test, ^****^P < .0001, three repeats, *n* = 95). **b** Silencing of *NbLecRK-IV.1* by VIGS construct (TRV-NbLecRK-IV.1) significantly reduces *P. infestans* colonization on *N. benthamiana* leaves expressing each construct as indicated in representative leaf images (under UV light). Mean lesion diameter after *P. infestans* inoculation was measured 5 days later (two-tailed *t*-test, ^****^P < .0001, *n* = 175). **c** Representative images showing disease lesions on StLecRK-IV.1 RNAi and control E3 potato leaves 5 days after inoculation with *P. infestans* isolate HB09-14-2. **d** Lesion sizes on StLecRK-IV.1-Ri11, StLecRK-IV.1-Ri13, StLecRK-IV.1-Ri20, and E3 control potato leaves at 5 dpi. These tests were carried out at least three times. Each experiment included at least 15 leaves from four independent plants. One-way ANOVA was used for statistical analysis (^*^P < .05, ^**^P < .01). Bars show mean lesion sizes ± standard error. **e** Significant reduction of sporangia number collected from *P. infestans*-infected StLecRK-IV.1-Ri potato leaves compared with E3 control. ^*^P < .05 indicates a significant difference (one-way ANOVA). StLecRK-IV.1 interacts with tetraspanin protein StTET8.

Several *Arabidopsis* LecRKs have been implicated in plant immunity [7–9, 14, 15, 34]. As *StLecRK-IV.1* responds to *P. infestans* infection (Fig. 1c), we further investigated the potential role of StLecRK-IV.1 in regulating defense against *P. infestans*. StLecRK-IV.1-GFP was transiently expressed in *N. benthamiana* leaves, which were then inoculated with *P. infestans* isolate 88069. The expression of StLecRK-IV.1-GFP in *N. benthamiana* leaves was confirmed by western blot and fluorescence microscopy (Supplementary Data Fig. S2A). At 5 days post-inoculation (dpi), *N. benthamiana* leaves with overexpression of *StLecRK-IV.1* had significantly increased *P. infestans* colonization with bigger lesion diameters compared with the control (Fig. 2a). On the contrary, we performed virus-induced gene silencing (VIGS) to knock down the expression of *NbLecRK-IV.1* (NbS00015931g0001.1) in *N. benthamiana* (Supplementary Data Fig. S2B and C). qRT–PCR was carried out to confirm VIGS silencing efficiency. Compared with the control TRV-GFP plants, TRV-NbLecRK-IV.1 plants showed a 75% reduction in the expression of *NbLecRK-IV.1* (Supplementary Data Fig. S2E). It is worth noting that silencing *NbLecRK-IV.1* by VIGS had no effect on plant development and growth in terms of plant size, leaf color, and leaf morphology (Supplementary Data Fig. S2D). *P. infestans* 88069 was inoculated in the VIGS plants. Compared with control, VIGS of *NbLecRK-IV.1* resulted in markedly smaller disease lesions at 5 dpi (Fig. 2b). These results suggested a negative role of *LecRK-IV.1* in plant resistance to *P. infestans.*

To further verify the role of *LecRK-IV.1* in potato resistance, stable RNAi transgenic potato lines were created to silence *StLecRK-IV.1* in potatoes (Supplementary Data Fig. S3). Three transgenic potato lines (lines 11, 13, and 20) with a significantly reduced expression level of *StLecRK-IV.1* were selected for inoculation of *P. infestans* isolate HB09-14-2. Consistent with the lesion reduction observed on *NbLecRK-IV.1* VIGS *N. benthamiana* plants, the StLecRK-IV.1 RNAi lines showed a significantly smaller lesion area (*P <* .05 or *P* < 0.01) (Fig. 2c and d) and fewer sporangia compared with the control E3 (Fig. 2e) at 5 dpi. There was no positive correlation between silencing efficiency and resistance (Fig. 2c–e; Supplementary Data Fig. S3D). Similar to NbLecRK-IV.1 VIGS plants, no discernible differences in growth or morphology between the RNAi lines and the E3 controls were observed (Supplementary Data Fig. S3C), implying that this gene may have only a small role in development.

Taken together, the results of transient expression of the *StLecRK-IV.1*, VIGS of *NbLecRK-IV.1*, and *StLecRK-IV.1* RNAi transgenic potato lines support the role of StLecRK-IV.1 and NbLecRK-IV.1 in negatively regulating resistance against *P. infestans* in these solanaceous plants.

**Figure 3:**
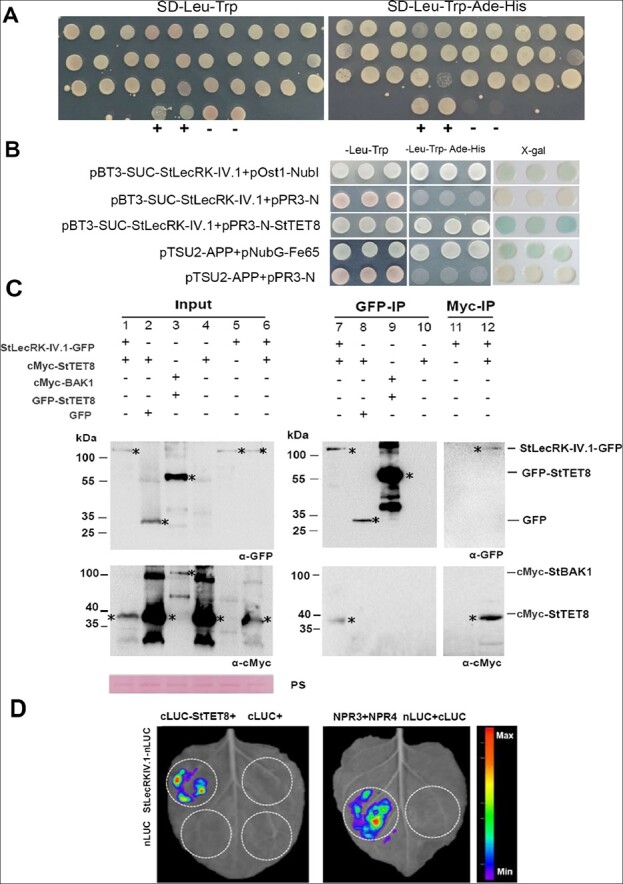
StLecRK-IV.1 interacts with StTET8 in yeast and *in planta*. **a** Potential StLecRK-IV.1 interaction yeast clones were screened by the DUALmembrane system. Four yeast clones in the last row in each picture are controls: +, positive control, −, negative control. **b** StLecRK-IV.1 interacts with StTET8 in yeast pairwise interaction assay. First row: the positive control combination indicates that pBT3-SUC-StLecRK-IV.1 was fully expressed, and the Cub moiety is accessible to interact with the Ost1-NubI moiety expressed by pOst-NubI control prey. Second row: negative control combination. pBT3-SUC-StLecRK-IV.1 does not interact with the Nub-fused nonsense-peptide expressed by pPR3-N control prey. Third row: pBT3-SUC-StLecRK-IV.1 interacts with pPR3-N-StTET8. Fourth row: positive control. Fifth row: negative control; pink color indicates negative interaction. **c** Co-immunoprecipitation assays confirmed the interaction of StTET8 and StLecRK-IV.1 *in planta*. Following pull-downs with GFP-trap beads, StLecRK-IV.1-GFP associated with cMyc-StTET8 (lane 7) but EV-GFP-did not (lane 8). GFP-trap beads could not immunoprecipitate cMyc-StTET8 alone (lane 10). GFP-StTET8 could not pull-down cMyc-StBAK1 (lane 9). Following pull-downs with cMyc-trap beads, cMyc-StTET8 immunoprecipitated StLecRK-IV.1-GFP (lane 12) but cMyc-trap beads could not pull down StLecRK-IV.1-GFP (lane 11). Expression of constructs in *N. benthamiana* leaves is indicated by a + sign. Protein size markers are indicated in kilodaltons (kDa), and protein loading is indicated by Ponceau stain (PS). Three additional repeats are shown in Supplementary Data Figure 5A. **d** The luciferase complementation assay confirmed that StLecRK-IV.1 interacts with StTET8. The figure shows the luminescence signal on *N. benthamiana* leaves collected by the plant live imager. White circles indicate the agroinfiltrated areas. Co-expression of StLecRK-IV.1-nLUC with cLUC-StTET8 shows a strong fluorescence signal, while the negative control StLecRK-IV.1-nLUC with cLUC, nLUC with cLUC-StTET8, or nLUC with cLUC shows no fluorescence signal. The combination of NPR3 and NPR4 is the positive control.

To identify putative interacting proteins of StLecRK-IV.1 involved in regulating the plant’s resistance, a yeast-two-hybrid (Y2H) screen was performed against a potato DUALmembrane system-based Y2H library with cDNAs generated from potato leaf material inoculated with *P. infestans* (Fig. 3a). The split-ubiquitin mechanism [35, 36] is used by the DUALmembrane system to detect the interaction between an integral membrane protein and its interaction partners. A total of 132 potential candidate interacting proteins were obtained, and then 10 candidate interacting proteins of interest were selected through consulting the literature and bioinformatics analysis for further verification. The DUALmembrane pairwise interaction assay finally confirmed three StLecRK-IV.1 interacting proteins: tetraspanin-8-like (corresponding to potato XP_006343564.1, hereafter referred to as StTET8), aquaporin PIP2-1 (XP_006357563.1), and outer envelope protein 61 (XP_006361116.1) (Fig. 3b). StTET8 shares high protein similarity with AtTET8 (Supplementary Data Fig. S4A and B), which is a tetraspanin and plays a crucial role in plant immunity [28]. Hence, we focused on StTET8 for further study.

The interaction between StLecRK-IV.1 and StTET8 was further confirmed *in planta* by co-immunoprecipitation conducted using *Agrobacterium*-mediated transient expression of protein fusions in *N. benthamiana*. Expression of each target protein was confirmed by western blot using α-GFP and α-cMyc antibodies in input samples (Fig. 3c, lanes 1–6). Following pull-downs with GFP-trap beads, StLecRK-IV.1-GFP was specifically associated with cMyc-StTET8 (Fig. 3c, lane 7) but control EV-GFP did not (Fig. 3c lane 8). GFP-trap beads could not immunoprecipitate cMyc-StTET8 alone (Fig. 3c lane 10), and GFP-StTET8 could not co-immunoprecipitate a control membrane protein, cMyc-BAK1 (Fig. 3c lane 9). In addition, following the incubation of samples with cMyc-trap beads, cMyc-StTET8 co-immunoprecipitated StLecRK-IV.1-GFP (Fig. 3c lane 12), but cMyc-trap beads did not pull down StLecRK-IV.1-GFP (Fig. 3c lane 11). The results showed that StTET8 specifically interacts with StLecRK-IV.1 but not with control membrane protein StBAK1 (Fig. 3c; Supplementary Data Fig. S5A).

A luciferase complementation assay was also performed to confirm their interaction. The combination of NPR3 and NPR4 was used as positive control [37]. The results showed that the luciferase signal could be detected in the infiltration area where StLecRK-IV.1-nLuc and cLuc-StTET8 were co-expressed rather than the control infiltrated areas (Fig. 3d; Supplementary Data Fig. S5B). To provide more evidence for StLecRK-IV.1 and StTET8 interaction, a bimolecular fluorescence complementation (BiFC) test was also conducted. cYFP-StLecRK-IV.1 was co-expressed with nYFP-StTET8, nYFP (N terminus-encoding portions of yellow fluorescent protein), or nYFP-StP2-like (pathogenesis-related protein P2-like precursor) by agroinfiltration in *N. benthamiana* leaves. The results showed that only the combination of cYFP-StLecRK-IV.1 and nYFP-StTET8 exhibited yellow fluorescence on the cell membrane, indicating their interaction in the plant cell (Supplementary Data Fig. S5C). Taken together, all these results demonstrate that StLecRK-IV.1 interacts with StTET8 both in yeast and *in planta*.

### Overexpression of StTET8 reduces *P. infestans* leaf colonization and StLecRK-IV.1 attenuates StTET8 function

**Figure 4:**
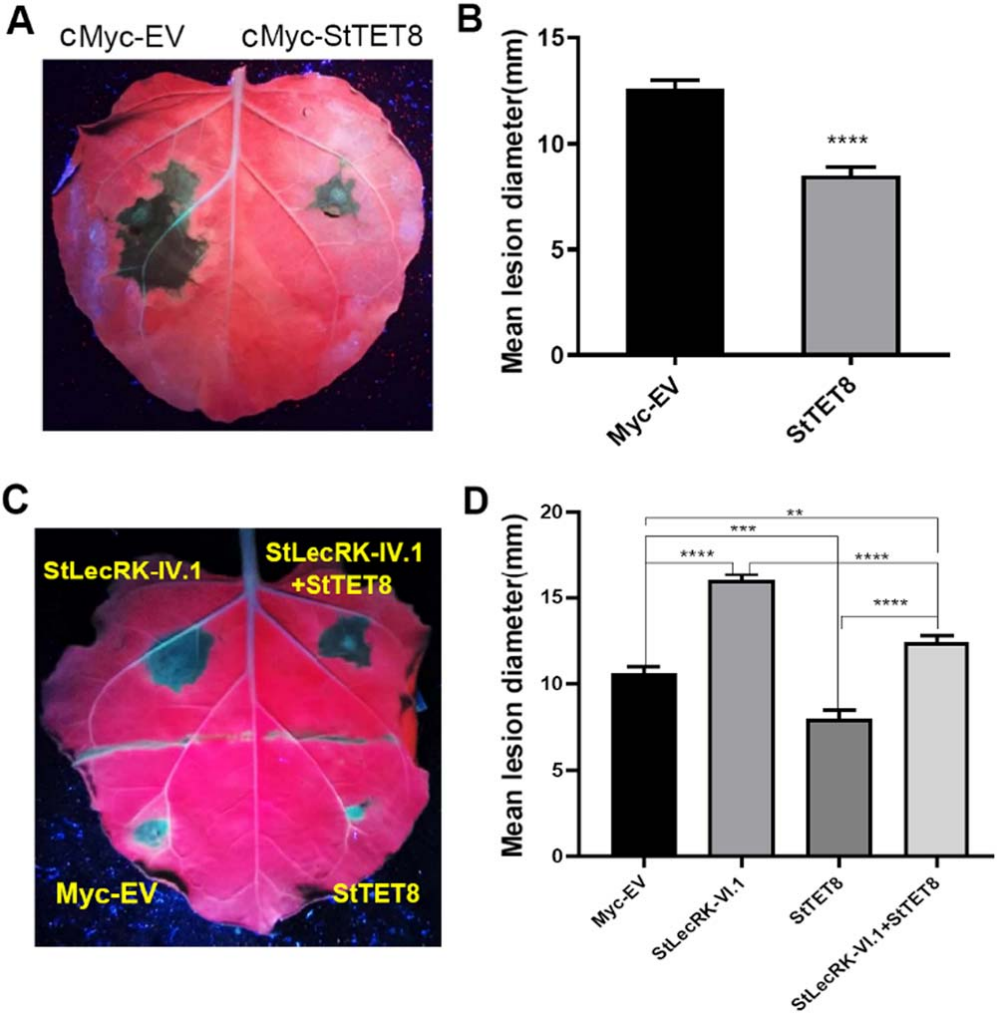
Transient overexpression of StTET8 reduces *P. infestans* leaf colonization, while StLecRK-IV.1 attenuates its function. **a** Demonstrative leaf image (under UV light) shows *P. infestans* lesions on *N. benthamiana* leaves transiently expressing cMyc-StTET8 and cMyc-EV. **b** Graph showing measurement of lesion diameter on *P. infestans* 88069-inoculated leaves (two-tailed *t*-test, ^****^P < .0001, three repeats, *n* = 90); values are mean ± standard error. **c** Image taken under UV light at 6 dpi (left) and **d** measurement of lesion diameter; transient overexpression of StTET8 improved disease resistance of *N. benthamiana* against *P. infestans* compared with the EV control, whereas co-expression of StLecRK-IV.1 with StTET8 significantly increased disease lesions versus StTET8, indicating that StLecRK-IV.1 attenuated StTET8 positive function in disease defense. Experiments were repeated three times. Statistical significance was determined using one-way ANOVA (^**^P < .01, ^***^P < .001, ^****^P < .0001, *n* = 60). Data represent the mean ± standard error.

As StLecRK-IV.1 negatively regulates plant immunity to *P. infestans* (Fig. 2), we transiently overexpressed its interacting protein, StTET8, in *N*. *benthamiana* leaves (Supplementary Data Fig. S4C) to test the function of StTET8 in the immune response. Compared with the cMyc-EV control, transient overexpression of *cMyc-StTET8* in *N. benthamiana* leaves resulted in considerably (*P* < .0001, two-tailed *t*-test) reduced *P. infestans* colonization (Fig. 4a and b). This finding showed that StTET8 is a positive regulator of *P. infestans* immunity.

The contrasting roles of StLecRK-IV.1 and StTET8 in resistance to *P. infestans* led us to examine whether they had an antagonistic effect when they were co-expressed. Empty vector (cMyc-EV), StLecRK-IV.1, StTET8, and StLecRK-IV.1 + StTET8 were transiently expressed in different parts of the same *N. benthamiana* leaf (Fig. 4c). Twenty-four hours after agroinfiltration, *P. infestans* 88069 was inoculated on each part, and lesion areas were measured 5 dpi. Again, the results confirmed that StLecRK-IV.1 enhanced and StTET8 restricted leaf colonization of *P. infestans* compared with cMyc-EV (Fig. 4d). Interestingly, when they were co-expressed, the immune function of each was attenuated significantly compared with expressing them alone (Fig. 4d). The lesion diameters with co-expression were smaller than those with StLecRK-IV.1 expression (Fig. 4d), suggesting that StTET8 partially antagonized the StLecRK-IV.1 function. The lesion diameters with co-expression were bigger than that of cMyc-EV control (Fig. 4d), indicating that StLecRK-IV.1 negative regulatory function overwhelmed the positive regulatory function of StTET8 but was compromised by overexpressed StTET8 to some extent.

### StTET8-associated vesicles accumulate around *P. infestans* hyphae

StTET8 shares 69% amino acid identity with AtTET8, and both contain four transmembrane domains and the same topology structures (Supplementary Data Fig. S4A and B), indicating they may share similar functions. AtTET8-associated vesicles accumulated at the infection sites of the fungal pathogen *B. cinerea* [28]. Thus, we tested whether StTET8 performs similar functions in potato upon the oomycete pathogen *P. infestans*.

**Figure 5:**
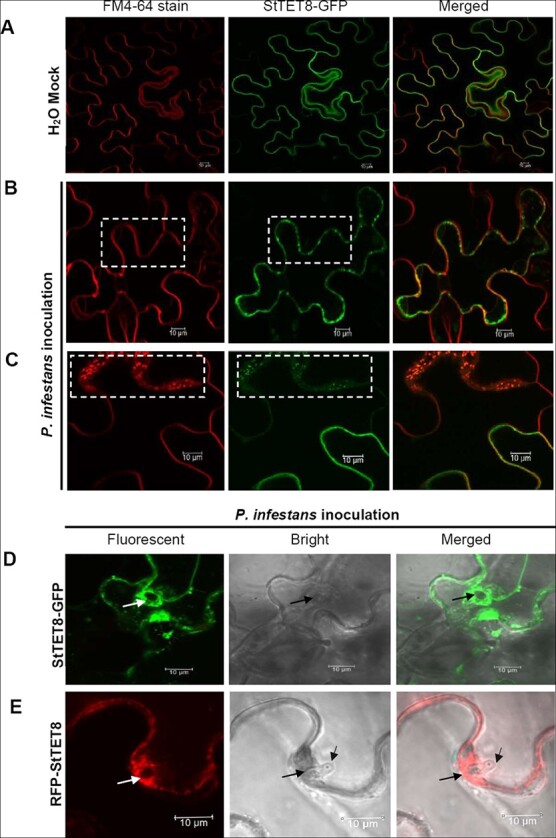
*P. infestans* triggers the accumulation of StTET8-associated vesicles around infection hyphae. **a** Images showing StTET8-GFP localization without *P. infestans* inoculation. The cell membrane was stained by FM4-64, showing red fluorescence. FM4-64 staining was conducted by incubation in 8 μM dye for 20 minutes. **b** Images showing StTET8-associated vesicles induced by *P. infestans* around the cell membrane (dashed rectangular areas). The images were captured by confocal microscopy 3 days after *P. infestans* inoculation. **c** Images showing that StTET8-associated exosomes might be transferred into extracellular areas (dashed rectangular areas). Agroinfiltration was used to transiently express StTET8-GFP in *N. benthamiana* leaves, following inoculation of *P. infestans* strain 88069 1 day later, and cells were visualized by confocal microscopy 3 days after *P. infestans* inoculation. **d**, **e** Images taken around the infection site of *P. infestans* show different sizes of StTET8-associated vesicles accumulated around hyphae. Both C-terminal GFP-labeled StTET8 (StTET8-GFP), shown in (**d)**, and N-terminal RFP-labeled StTET8 (RFP-StTET8), shown in (**e**) StTET8-associated vesicles (green or red fluorescence-labeled), accumulated around the intracellular hyphae of *P. infestans*. Arrows show infection hyphae. Scale bar represents 10 μm.

When StTET8-GFP was expressed by agroinfiltration in *N. benthamiana* leaves, clear green fluorescence was observed around the cell membrane, which was reflected by plasma membrane marker FM4-64 staining of the cell membrane (red channel) (Fig. 5a; Supplementary Data Fig. S6A and B). This is consistent with the result reported by Cai *et al*. [28]. After *P. infestans* inoculation, many there were many StTET8-associated vesicles near the cell membrane, as indicated by the presence of numerous GFP-labeled vesicles (Fig. 5b; Supplementary Data Fig. S6C). In addition, we also observed the presence of StTET8-associated vesicles in the extracellular matrix (Fig. 5c), which might be transferred into the extracellular matrix as cargoes when challenged by *P. infestans*. Furthermore, we observed that StTET8-associated vesicles accumulated at the infection site around the invaded *P. infestans* hyphae rather than the control leaves (mock treatment with water) (Fig. 5a and d; Supplementary Data Fig. S6A and D). This is also consistent with the observation of AtTET8-associated vesicles responding to *B. cinerea* infection [28]. Similar results were obtained when red fluorescent protein (RFP) was used to label StTET8 (RFP-StTET8) (Fig. 5e; Supplementary Data Fig. S6E). These data revealed that StTET8-associated vesicles responded to *P. infestans* challenge and accumulated around the *P. infestans* infection sites.

### StLecRK-IV.1 affects StTET8 stability

**Figure 6:**
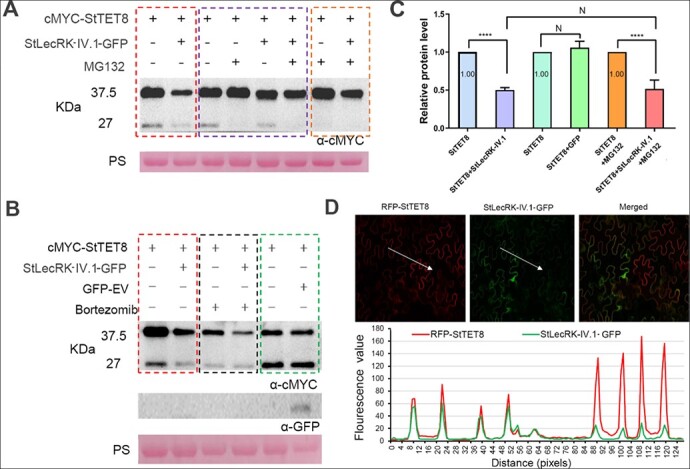
StLecRK-IV.1 affects the protein stability of StTET8. **a** Western blot showing that co-expression of StTET8 with StLecRK-IV.1 leads to reduction of StTET8 compared with StTET8 expression alone in the absence of MG132 (emphasized by the dashed red rectangle) or presence of MG132 (dashed orange rectangle). MG132 did not affect StTET8 and StLecRK-IV.1 expression level (dashed purple rectangle). **b** Western blot showing that bortezomib did not inhibit the reduction of StTET8 triggered by StLecRK-IV.1 (dashed black rectangle), and co-expression of GFP with StTET8 did not trigger StTET8 reduction (dashed green rectangle). Transient expression was performed by agroinfiltration in *N. benthamiana* leaves. Western blots were conducted using cMyc and GFP antibodies. The construct’s expression in leaves is denoted by +. KDa indicates protein size markers, and protein loading was marked by Ponceau stain (PS). Additional Western blot repeats are found in the Supplementary Data File. **c** Graph showing relative StTET8 protein levels when co-expressed with different partners. The results demonstrated that StLecRK-IV.1 led to a reduction in StTET8, reflected by a weak western blot band, but not by GFP control. To quantify cMyc-StTET8 protein level, each western blot band intensity of cMyc-StTET8 was normalized by the Ponceau stain band signal. Data collected from three or four independent repeats (from panels **a** and **b**; Supplementary Data Fig. S7) were combined and statistically analyzed. **d** Images showing that stronger expression of GFP-StLecRK-IV.1 leads to weak fluorescence intensity of RFP-StTET8 in the same plant cell, indicating that StLecRK-IV.1 affects the stability of StTET8 in plant cells. Protein transient expression was performed by agroinfiltration in *N. benthamiana* leaves.

The attenuation of the positive immune function of StTET8 by StLecRK-IV.1 (Fig. 4) prompted us to investigate whether StTET8 protein stability was affected by StLecRK-IV.1. To test this hypothesis, *N. benthamiana* leaves were transiently co-expressed with StLecRK-IV.1-GFP and cMyc-StTET8. The results showed that co-expression of cMyc-StTET8 with StLecRK-IV.1-GFP led to reduction of cMyc-StTET8 protein level (Fig. 6a and b; Supplementary Data Fig. S7A, C, and D, dotted red rectangle). However, StLecRK-IV.1-GFP did not affect cMyc-GUS stability compared with GFP-EV control (Supplementary Data Fig. S7C, dotted blue rectangle, three repeats).

Reduction of stability of cMyc-StTET8 by StLecRK-IV.1-GFP could be due to the function of proteasomes. We used proteasome inhibitors MG132 (for 26S) and bortezomib (for 20S) to test this theory. The results showed that the presence of MG132 or bortezomib did not prevent the cMyc-StTET8 protein reduction when it was co-expressed with StLecRK-IV.1-GFP (Fig. 6a and b; Supplementary Data Fig. S7A and B, dotted orange rectangle). Interestingly, there was a 27-kDa band accompanied by a 37.5-kDa band when cMyc-StTET8 was expressed without MG132 (Fig. 6a, dashed red rectangle; Supplementary Data Fig. S7D), but a very weak 27-kDa band appeared when MG132 was added [Fig. 6a (purple rectangle) and b].

To quantify cMyc-StTET8 protein levels, each western blot band intensity of cMyc-StTET8 was normalized by the Ponceau stain band signal. Data from three or four independent repeats (Fig. 6; Supplementary Data Fig. S7) were combined and statistically analyzed. As shown in Fig. 6c, StLecRK-IV.1-GFP significantly affected the protein stability of cMyc-StTET8 when they were co-expressed in a condition with or without MG132. GFP-EV did not affect cMyc-StTET8 stability when they were co-expressed in *N. benthamiana* leaves, demonstrating that StLecRK-IV.1 specifically affected the stability of cMyc-StTET8.

To provide additional evidence, StLecRK-IV.1-GFP and RFP-StTET8 were co-expressed in *N. benthamiana* leaf. Confocal microscope observation showed that when they were co-expressed in the same cell, stronger red fluorescence on the membrane was accompanied by weak green fluorescence, while weak red fluorescence on the membrane was accompanied by stronger green fluorescence (Fig. 6d). However, co-expressing GFP with RFP-StTET8 did not affect RFP-StTET8 red fluorescence intensity (Supplementary Data Fig. S7E). These data further confirmed that StLecRK-IV.1 affects the stability of StTET8 in plant cells. Taken together, both the western blot and fluorescent microscope observation results strongly support the idea that StLecRK-IV.1 affects StTET8 stability.

## Discussion

Plant LecRKs have been reported to play vital roles in plant development as well as in plant responses to biotic and abiotic stressors [4, 38, 39]. Multiple studies have revealed that plant LecRKs are involved in plant immunity against various pathogens, including bacteria, fungi, and oomycetes. Most reported LecRKs positively regulate plant immunity [5]. Some of them play a dual role in controlling different pathogen defenses. Overexpressing *LecRK-V.5* in *Arabidopsis* leads to more resistance to *Phytophthora capsica* but higher susceptibility to *P. syringae* pv. *tomato* DC3000 [15]. Phylogenetic analysis showed that AtLecRK-IV.3 and StLecRK-IV.1 are located in the same clade (clade IV) (Supplementary Data Fig. S1). AtLecRK-IV.3 (AtLPK1) has been reported to positively regulate *Arabidopsis* resistance against *B. cinerea* infection [14]. Our results showed that StLecRK-IV.1 is a negative regulator of plant immunity, as RNAi of *StLecRK-IV.1* in potato or VIGS silencing of *NbLecRK-IV.1* in *N. benthamiana* showed enhanced resistance against *P. infestans*, while transient expression of *StLecRK-IV.1* in *N. benthamiana* led to plants being more susceptible to *P. infestans* (Fig. 2). Some LecRKs have been reported to be involved in plant development [40–42]. In the present study, however, StLecRK-IV.1 RNAi potato lines and VIGS of *NbLecRK-IV.1* in *N. benthamiana* plants showed no obvious developmental alterations. It would be an appropriate target gene for potato late blight resistance improvement by a knockout strategy using gene-editing technologies such as the CRISPR-Cas9 system, as shown by Kieu *et al*. [43].

Many *LecRK* genes respond to different biotic and abiotic stresses, which is crucial for the activation of their function. Our findings revealed that *StLecRK-IV.1* expression was suppressed by *P. infestans* at an early stage. Upon *P. infestans* infection, *StLecRK-IV.1* was downregulated at 24 h (Fig. 1c), which is in agreement with Zhang *et al*. [30] (refer to potato transcript PGSC0003DMP400015651). According to Zhang *et al*. report [30], *StLecRK-IV.1* also responds to *P. carotovorum* ssp. *brasiliense* (*Pcb*), meaning that it may engage in more pathogen resistance. In addition, *StLecRK-IV.1* was significantly induced by ABA, which indicated that StLecRK-IV.1 might be involved in abiotic stress. It has been reported that *LecRK* is regulated by ABA and involved in stomatal closure [15, 44]. Interestingly, one of the StLecRK-IV.1-interacting proteins, StPIP2-1, is an aquaporin protein, which is considered to be related to stoma regulation and associated with ABA [45, 46]. Therefore, it would be interesting to investigate whether StLecRK-IV.1 is engaged in ABA-mediated stomatal immunity and abiotic stress response in potatoes.

TETs have been proven to play crucial roles in regulating plant immunity [28]. In *Arabidopsis*, *tet8*/*tet9* double mutant lines showed enhanced susceptibility to *B. cinerea.* TET8-associated vesicles accumulated at the sites of fungal infection and were involved in enhancing resistance to the fungus by taking the host small RNAs (sRNAs) into fungal cells to inhibit the invasion [28]. In this study, we found that StTET8, an ortholog of *Arabidopsis* TET8, interacts with StLecRK-IV.1. Transient overexpression of *StTET8* in *N. benthamiana* reduces *P. infestans* colonization, demonstrating StTET8 is a positive plant immunity regulator (Fig. 4). In accordance with TET8-associated vesicles accumulating at the sites of *B. cinerea* infection, we also found that StTET8-associated vesicles accumulated at the sites of *P. infestans* infection around the hyphae (Figs 5d and e; Supplementary Data Fig. S6D and E), suggesting that StTET8 is involved in host immunity in the same manner as AtTET8. In the battle between plants and pathogens, fungal pathogens transfer sRNAs into host plants to silence plant immunity genes [47]. On the contrary, host plants deliver sRNAs into fungal cells to inhibit the expression of genes related to fungal virulence [28, 48]. There is no report showing that host plants transfer sRNAs into *P. infestans* cells to suppress colonization at present; however, our results indicated that potato plants might share the same mechanism to combat the oomycete pathogen *P. infestans*.

LecRKs utilize different mechanisms to manipulate plant immunity. LecRLKs contain a Ser/Thr kinase domain that confers Ser/Thr kinase activity [49]. Some LecRLKs may appear as dual activities of both Ser/Thr kinase and Tyr kinase [50]. It has been reported that LecRK-VI.2 fulfills its function by forming a complex with FLS2 to activate the MAPK signaling cascade and induce PTI marker genes such as *FRK1* and *WRKY53*, and control stomata closure during PTI [8, 51]. LecRK-I.9 detects extracellular ATP signals and phosphorylates RBOHD (Respiratory burst oxidase homolog protein D) directly, triggering Ca^2+^ influx, MAPK activation, ROS accumulation, and defense gene expression [10, 52, 53]. To regulate ABA-mediated stomatal movements, LecRK-VI.4 phosphorylates many proteins, closely related to stomatal function, involved in aquaporin activity, H^+^ pump activity, and the Ca^2+^ signaling pathways [34].

StTET8 acts as a positive plant immunity regulator (Fig. 4a and b), whose function was attenuated by the co-expression of StLecRK-IV.1 (Fig. 4c and d). Consistent with this, StTET8 protein stability is also reduced by co-expression of StLecRK-IV.1 (Fig. 6a and b). Considering that StLecRK-IV.1 contains a tyrosine kinase domain (Fig. 1), we speculate that StLecRK-IV.1 reduced the stability of StTET8 through the phosphorylation of StTET8; subsequently, the phosphorylated StTET8 was turned over by an unknown mechanism. It has been reported that tyrosine phosphorylation controls the transcriptional activity of CjWRKY1 by reducing binding activity and promoting its degradation to regulate benzylisoquinoline alkaloid biosynthesis by the proteasome [54]. Moreover, a *Phytophthora sojae* crinkler (CRN) effector has been found to mediate the phosphorylation and degradation of plant aquaporin proteins in order to reduce host immunity [55]. However, the 26S and 20S proteasome inhibitors MG132 and bortezomib did not inhibit the protein reduction of StTET8 caused by StLecRK-IV.1 (Fig. 6a and b), indicating that StLecRK-IV.1-triggered StTET8 degradation was independent of the 26S and 20S proteasomes. More biochemical and enzymatic studies are needed in the future to clarify the mechanism of how StLecRK-IV.1 affects the stability of StTET8.

As a potential cell surface receptor, future research will focus on its detailed regulation mechanisms, the ligand of StLecRK-IV.1, and how StLecRK-IV.1 perceives extracellular signals and transduces the perceived signals, and also what components are associated with and can be phosphorylated by StLecRK-IV.1. Overall, our evidence demonstrated that a potato StLecRK-IV.1 negatively regulates late blight resistance by interacting with and affecting the protein stability of a positive regulator, StTET8. This study provides a novel interaction mechanism of how a plant LecRLK regulates the immunity of plants.

## Materials and methods

### Plasmid constructs

The *StLecRK-IV.1* gene was cloned using a two-step PCR from potato line DM1-3 (which was used for potato genome sequencing) cDNA with gene-specific primers modified to contain the Gateway^®^ (Invitrogen) attB recombination sites. pDONR221 (Invitrogen) was used for recombination with the purified PCR product to generate entry clones. A Y2H membrane prey library was used to amplify the full length of StTET8. The entry clones were also generated in the same way. All the proteins were fused with a tag through combined entry clones with plant expression vectors as follows: pK7FWG2.0 [for C-terminal enhanced GFP (eGFP) fusion] or pK7WGR2 (for N-terminal RFP fusion). For luciferase complementation assays, *StLecRK-IV.1* and *StTET8* were cloned into pCAMBIA1300-Nluc/Cluc using *KpnI* and *SalI* sites to produce StLecRK-IV.1-nLUC and cLUC-StTET8, respectively. For split-YFP constructs, *StLecRK-IV.1* and *StTET8* were recombined with pCL113 (for N-terminal cYFP fusion) and pCL112 (for N-terminal nYFP fusion), respectively. pH7LIC 9.1 was produced utilizing the ClonExpress Entry One Step Cloning Kit (Vazyme) for N-terminal tagging. Primer sequences used in this research are shown in Supplementary Data Table S1.

### Potato transformation and plant growth conditions

The RNAi construct was designed at the 1657–1956 bp sites of *StLecRK-IV.1* and cloned into pHellsgate 8 (an expression vector) to generate pHellsgate8-35S-StLecRK-IV.1-RNAi interference vector, and *Agrobacterium tumefaciens* containing the pHellsgate8-35S-StLecRK-IV.1-RNAi vector was transformed into the potato cultivar ‘E-potato-3’ (E3) utilizing a microtuber disk as explant, as described by Tian *et al*. [56]. Putative transgenic potato plants harboring pHellsgate8-35S-StLecRK-IV.1-RNAi vector were first screened on differential medium [Murashige and Skoog medium (MS) + 30 g/l sucrose + 0.5 mg/l 6-benzylamino purine (6-BA) + 0.2 mg/l gibberellin A3 (GA3) + 0.2 mg/l indole-3 acetic acid (IAA) + 2 mg/l zeatin + 2.2 g/l Phytagel, pH 5.9], second shift to selective medium (MS + 30 g/l sucrose + 0.5 mg/l 6-BA + 0.2 mg/l GA3 + 0.2 mg/l IAA + 2 mg/l zeatin + 75 mg/l kanamycin + 400 mg/l cefotaxime + 2.2 g/l Phytagel, pH 5.9), and then transferred to root generation medium (MS + 50 mg/l kanamycin + 400 mg/l cefotaxime + 2.2 g/l Phytagel, pH 5.9). Positive lines were confirmed by RT–PCR, and further assessment of gene expression levels was performed by qRT–PCR. Positive potato plantlets were cultured at 23°C in a temperature-controlled environment (16 hours light and 8 hours dark). Then, the positive potato plants were transplanted into a greenhouse, and the leaves were used for experiments 8 weeks after transplantation. The *N. benthamiana* plants used were cultured in a controlled chamber at 22°C for 16 hours during the day and 8 hours at night. In different experiments, we chose appropriate *N. benthamiana* leaves of different seedling ages, between 3 and 5 weeks old in general (3–4 weeks old for BiFC and 4–5 weeks for *P. infestans* inoculation).

### Plant treatments and gene expression assay

Potato plant leaves were used when they reached 6 weeks old. A total of three leaves (from the top third to fifth compound leaf) from three separate plants were inoculated with *P. infestans* HB09-14-2. For each time point (0, 24, 48, 72 hours following *P. infestans* inoculation), three leaves were taken from individual plants and snap-frozen in liquid nitrogen. For plant defense hormone treatment, potato leaves were treated by spraying 1 mM ABA, 0.05 mM brassinolide (applied as epibrassinolide), 1 mM ethylene (applied as ACC), 1 mM salicylic acid, 1 mM methyl jasmonate and ddH_2_O. All solutions contained 1% dimethyl sulfoxide (DMSO). Six hours later three leaf disks (9 mm in diameter) were collected and frozen in liquid nitrogen. For RNA extraction we used the Plant Total RNA Kit (Zoman Biotechnologies, Beijing, China), and 5× All-In-One RT MasterMix (ABM) was used for the synthesis of the first-strand cDNA. Power SYBR Green was used for qRT–PCR experiments (Bio-Rad). The comparative ΔΔCt method was used for gene expression level analysis utilizing *StEF1α* as the reference gene for potato. Supplementary Data Table S1 lists the primer sequences.

### *P. infestans* isolates and the inoculation assay

*P. infestans* isolates 88069 and HB09-14-2 (race 1.2.3.4.5.6.7.9.10.11, collected from Hubei Province, China), were cultured and propagated with Rye Suc Agar plates for 14 days at 16°C in the dark. The former was mainly used to infect *N. benthamiana* leaves, and the latter was used to inoculate the leaves of potatoes. Petri dishes containing 14-day-old cultures were cleaned and diluted in sterile distilled water to collect sporangia. The spore concentration of the inoculum was 2 × 10^5^ ml^−1^ for *N. benthamiana* leaves (transient expression assay and VIGS leaves) and 8 × 10^4^ ml^−1^ for potato leaves. After a 2-hour incubation period at 4°C, the zoospores were harvested. Then, 10-μl droplets were inoculated on leaves of *N. benthamiana* or potato, which were put on moist paper towels in a sealed transparent box. The mean lesion diameter on leaves was assessed at 7 dpi, sporangia were counted at 10 dpi, and leaves from StLecRK-IV.1-RNAi potato plants were rinsed in 5 ml of H_2_O. All data analysis was performed by one-way ANOVA, and Graphpad Prism 7.0 software was used for pairwise or multiple comparisons. The error bars in the figures and all the values shown are averages ± standard deviations or standard errors of three or more replicates.

### Transient expression mediated by *Agrobacterium*

*Agrobacterium* strain GV3101 containing target construct was cultured in yeast extract beef medium with adequate antibiotics at 28°C and 200 rpm. After overnight culture, the cells were centrifuged at 4000 g for 10 minutes, and the pellet was resuspended in appropriate sterile 10 mM MES (4-Morpholineethanesulfonic acid), 10 mM MgCl_2_, and 200 mM acetosyringone. Before infiltrating *N. benthamiana* leaves, the culture was diluted to an ultimate OD_600_ (0.1 for infection assays, 0.05–0.1 for BiFC, and 0.5–1.0 for western blot).

### Bioinformatic analysis

NCBI (https://www.ncbi.nlm.nih.gov/) was used to retrieve the gene and amino acid sequences of *Solanum tuberosum* LecRK-IV.1, TET8 SMART software (http://smart.embl-heidelberg.de/), and NCBI conserved domains (https://www.ncbi.nlm.nih.gov/Structure/cdd/wrpsb.cgi) was used for protein structure and sequence analysis. The SignalP 5.0 server ((https://services.healthtech.dtu.dk/service.php?SignalP-5.0)) predicted the existence and location of signal peptides as well as their cleavage sites. Transmembrane domains were predicted using Phobius prediction (https://www.ebi.ac.uk/Tools/pfa/phobius/). The maximum likelihood approach and a JTT matrix-based model were used to infer the evolutionary history. The tree with the highest log likelihood (−54881.59) was shown. Initial tree(s) for the heuristic search were obtained automatically by applying Neighbor-Join and BioNJ algorithms to a matrix of pairwise distances estimated using a JTT model, and then selecting the topology with a superior log-likelihood value. MEGA X was used to undertake evolutionary analysis. ClustalX and Genedoc were used to align amino acid sequences between probable orthologs in different species.

### DUALmembrane system yeast-based screen assay

The DUALmembrane system uses the split-ubiquitin mechanism [36] to detect the interaction between membrane proteins (www.dualsystem.com). *StLecRK-IV.1* was cloned into pBT3-SUC vector to produce bait vector, and the bait plasmid was then co-transformed with the positive control plasmids pOst1-NubI and negative plasmids pPR3-N into the reporter strain NMY51, respectively. Using the selective plate (SD-trp-leu) to ensure that the bait was working properly in the DUALmembrane system, we screened the bait against a potato membrane yeast library [cDNA fused with NubG (pPR3-N)], and co-transformed pTSU2-APP with pNubG-Fe65, pTSU2-APP with pPR3-N into NYM51, respectively, were utilized as positive and negative control, respectively. The interaction between pBT3-SUC-StLecRK-IV.1 and its interactors was confirmed by pairwise interaction assay, and SD-leu-trp-his-ade with 5 mM 3-aminotriazole (3-AT) was used as a selective medium. Testing the activation of reporter genes in the X-gal assay was used to identify transformants. For each transformation, plasmid DNA (transformation reaction: 1.5 μg pBT3-SUC-StLecRK-IV.1 for bait transformation, 7 μg for screening library) and 100 μl denatured sheared salmon sperm DNA were mixed together with 2.5 ml PEG/liOAC mix and 600 μl yeast competent cells; PEG/liOAC mix contains 100 mM lithium acetate/10 × TE (Tris-EDTA) buffer pH 7.5/40% PEG 3350. Then all components were vortexed and incubated at 30°C for 45 minutes. A total of 160 μl DMSO was added, vortexed and heat-shocked at 42°C for 20 minutes, and centrifuged at 700 × g for 5 minutes; the cells were then resuspended in 3 ml 2 × YPDA (Yeast Peptone Dextrose Adenine) Medium and were recovered at 30°C for 90 minutes at 150 rpm, and centrifuged for 5 minutes at 700 × g. The pellet was resuspended in 4.8 ml 0.9% NaCl, plated on selective medium, and incubated for 4 days at 30°C.

### Confocal imaging

An *A. tumefaciens* strain GV3101 containing target constructs was infiltrated into the leaves of 3- to 4-week-old *N. benthamiana* plants. Using a confocal laser scanning microscope (Leica TCS-SPE), leaves expressing fluorescent protein fusions were observed and imaged at 2 days after agroinfiltration. The plasma membrane was stained with 8 μM FM4-64 dye (Invitrogen) for 20 minutes. GFP was stimulated at 488 nm with an argon laser, and its emissions were detected between 495 and 531 nm. Monomeric RFP and mOrange were stimulated at 561 nm and their emissions were detected at 600–630 nm. FM4-64-stained samples were stimulated at 514 nm and detected at a maximum of 640 nm. Split-YFP was stimulated at 514 nm and emissions were detected between 530 and 575 nm. To reduce ectopic protein expression artifacts, images were taken from leaf cells with medium to low levels of fluorescence.

### Luciferase complementation assay

The target plasmids were transformed into *A. tumefaciens* strain GV3101 and infiltrated into the leaves of *N. benthamiana*. cLUC-StTET8 was agroinfiltrated 1 day before StLecRK-IV.1-nLUC. Leaf samples were collected 2 days after the agroinfiltration of StLecRK-IV.1-nLUC. A total of 15 mM of luciferin was sprayed onto leaves, which were kept in the dark for 15 minutes, and the leaves were then detached to observe the fluorescence. The luciferase complementation assay images were captured using the NightShade LB 985 In Vivo Plant Imaging System. The experiment was repeated twice.

### Tobacco rattle virus-based VIGS *NbLecRK-IV.1* in *N. benthamiana*

The *N. benthamiana* database of Genome and Transcriptome (http://benthgenome.qut.edu.au/) and Solanaceae Genomics Network (https://solgenomics.net/) were used to identify the orthologs of *StLecRK-IV.1* in *N. benthamiana*. The region sharing low identity with other sequences in *N. benthamiana* was selected to make the VIGS construct. For the VIGS experiment, plasmids pTRV1 and pTRV2 were utilized [57]. The selected region of NbLecRK-IV.1 was amplified from *N. benthamiana* cDNA and inserted into pTRV2 vectors between *BamHI* and *EcoRI* sites in antisense orientation. A pTRV2 vector fused with GFP was used as a control [58]. *A. tumefaciens* harboring pTRV1 and pTRV2 gene vectors combined at a 1:1 ratio with OD_600_ 0.3 were injected into a four-leaf-stage *N. benthamiana*. *NbLecRK-IV.1* silencing efficiency was checked by qRT–PCR 2 or 3 weeks after infiltration and afterwards used for *P. infestans* inoculation. Three biological duplicates were performed in each assay.

### Co-immunoprecipitation and western blot

StLecRK-IV.1-GFP with cMyc-StTET8, cMyc-StBAK1 with GFP-StTET8, GFP with cMyc-StTET8, cMyc-StTET8 alone, and StLecRK-IV.1-GFP alone were agroinfiltrated into the leaves of 4- or 5-week-old *N. benthamiana* plants. Leaves were harvested at 2 dpi, and membrane protein was extracted using the Minute™ Plasma Membrane Protein Isolation Kit for Plants (SM-005-P, Invent). Using GFP Agarose Beads (KTSM1301, AlpaLife) and cMyc Agarose Beads (KTSM1306, AlpaLife), GFP-tagged and cMyc-tagged StLecRK-IV.1/TET8 fusions were immunoprecipitated. Sodium dodecyl sulfate–polyacrylamide gel electrophoresis (SDS–PAGE) was used to separate the obtained samples. Immunoprecipitated GFP or cMyc fusions and co-immunoprecipitated cMyc or GFP fusions were detected using an appropriate antibody. For the StTET8 degradation assay, by agroinfiltration with an *A. tumefaciens* strain (GV3101) containing corresponding constructs, target proteins were transiently expressed in *N. benthamiana* leaves. Two days later, four leaf disks (9 mm in diameter) were harvested, frozen in liquid nitrogen, and pulverized into powder. MG132 and bortezomib (40 μM) were infiltrated into leaves 8 h before samples were collected. Four hundred microliters of extraction buffer (10% glycerol, 25 mM Tris–HCL pH 7.5, 300 mM NaCl, 1 mM EDTA) with 10 mM dithiothreitol (DTT), 0.2% Nonidet P40, 1 mM phenylmethylsulfonyl fluoride, and protease inhibitor cocktail (Sigma) were added to each sample for protein extraction, then the samples were placed on ice for 30 minutes to thaw completely, and vortexed briefly for 10 minutes. Then, the samples were centrifuged at 13 000 rpm for 10 minutes at 4°C, and the supernatant was transferred to a precooled Eppendorf tube. The samples were then incubated for 10 minutes at 95°C in 2 × SDS buffer supplemented with 200 mM DTT. SDS–PAGE was used to separate the samples, which were then transferred to a PVDF membrane for western blot analysis. The conditions for western blotting were according to Ren *et al*. [59]. The relative protein quantification in the graph was calculated by Image J software.

## Acknowledgements

We are grateful for financial support from the National Natural Science Foundation of China (grant numbers 31761143007, 32072121, and 31471550).

## Author contributions

Z.T. and W.L. conceived the research and designed the experiments. L.G. conducted most of the experiments. Y.Q. performed VIGS and made the plants. Y.M. performed the partial Y2H screen and constructed the plasmids. J.Z. performed pathogen assays. L.G. and Z.T. performed data analysis and made the figures. L.G., W.L. and Z.T. wrote the paper with contributions from all the authors. Z.T. secured funding. All authors contributed to the final version of the manuscript.

## Data availability

All data are available in the manuscript or the supplementary material.

## Conflict of interest

All authors declare no conflict of interests.

## Supplementary data

Supplementary data is available at *Horticulture Research* online.

## Supplementary Material

Web_Material_uhac010
