# Supplementary material for: Potato StLecRK-IV.1 negatively regulates late blight resistance by affecting the stability of a positive regulator StTET8
Source: Hortic Res. 2022 Feb 11;9:uhac010. doi: 10.1093/hr/uhac010 (PMC9016858; doi:10.1093/hr/uhac010)
Supplement: Web_Material_uhac010 [file web_material_uhac010.pdf]

Figure S1

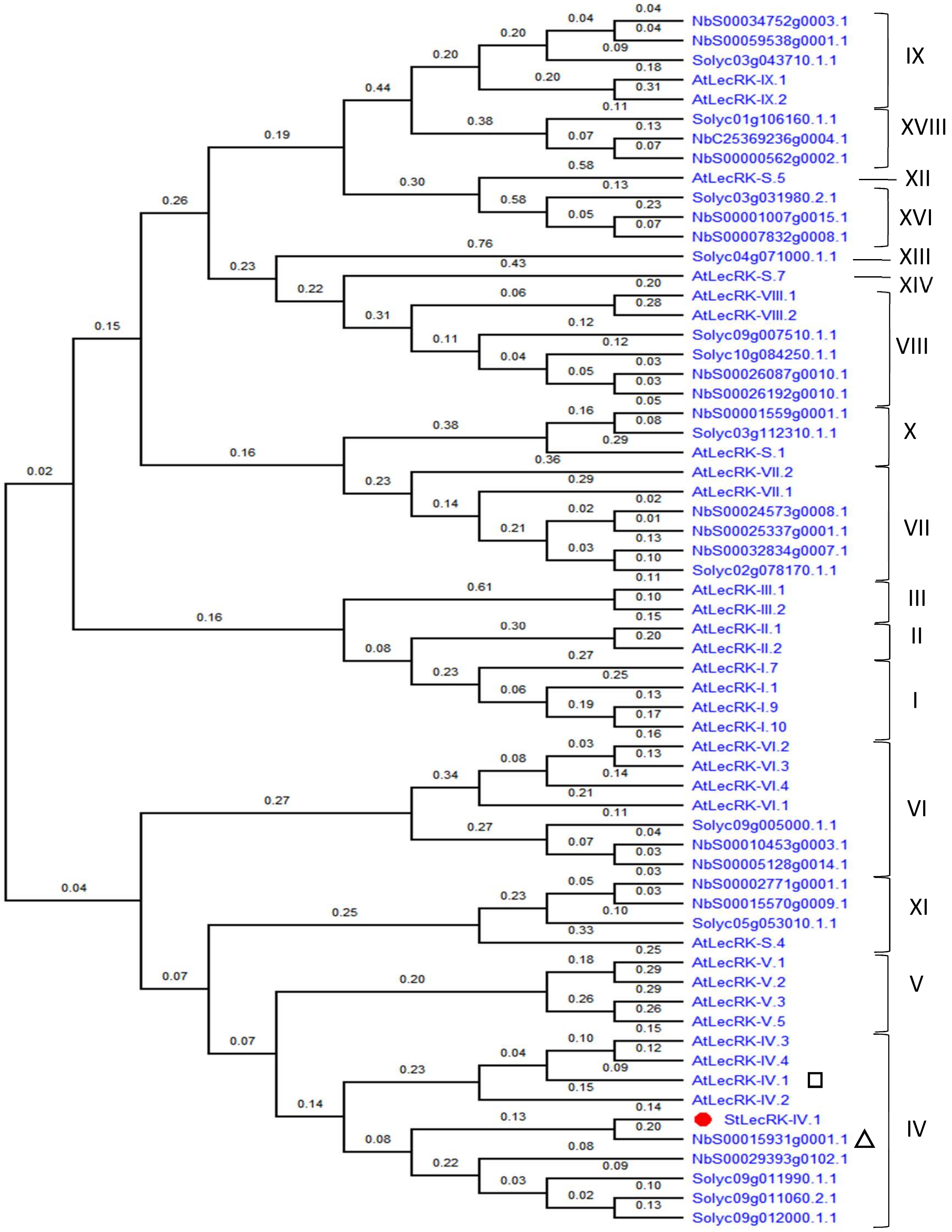

Supplementary Figure 1. Phylogenetic tree of LecRLK family from Solanaceous plants *N. bentheminana*, *Solanum lycopersicum*, and model plant *Arabidopsis*. All the amino sequence are obtained by NCBI BlastP. Red dot indicates the target protein: StLecRK-IV.1. □ and Δ indicates StLecRK-IV.1 orthologue in *Arabidopsis* and *N. benthemiana*.

Figure S2

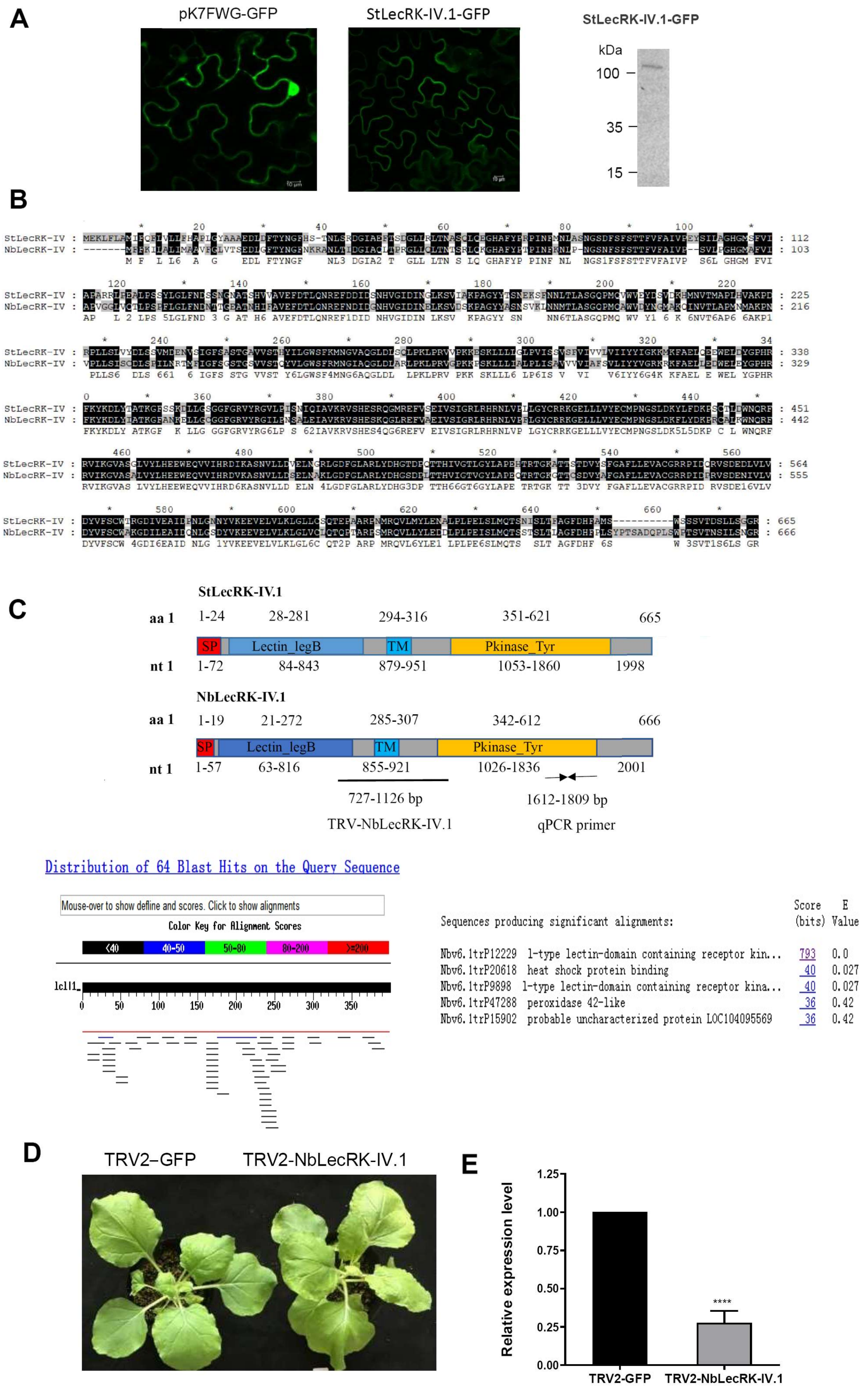

A
pK7FWG-GFP
StLecRK-IV.1-GFP
StLecRK-IV.1-GFP
kDa
100
35
15
B
StLecRK-IV
NbLecRK-IV
C
StLecRK-IV.1
aa 1
1-24
28-281
294-316
351-621
665
SP
Lectin_legB
TM
Pkinase_Tyr
nt 1
1-72
84-843
879-951
1053-1860
1998
NbLecRK-IV.1
aa 1
1-19
21-272
285-307
342-612
666
SP
Lectin_legB
TM
Pkinase_Tyr
nt 1
1-57
63-816
855-921
1026-1836
2001
727-1126 bp
TRV-NbLecRK-IV.1
1612-1809 bp
qPCR primer
Distribution of 64 Blast Hits on the Query Sequence
Mouse-over to show defline and scores. Click to show alignments
Color Key for Alignment Scores
<40
40-50
50-80
80-200
>=200
lcl1l
0
50
100
150
200
250
300
350
Sequences producing significant alignments:
Score (bits)
E Value
Nbv6.1trP12229 l-type lectin-domain containing receptor kin... 793 0.0
Nbv6.1trP20618 heat shock protein binding 40 0.027
Nbv6.1trP9898 l-type lectin-domain containing receptor kina... 40 0.027
Nbv6.1trP47288 peroxidase 42-like 36 0.42
Nbv6.1trP15902 probable uncharacterized protein LOC104095569 36 0.42
D
TRV2-GFP
TRV2-NbLecRK-IV.1
E
Relative expression level
1.25
1.00
0.75
0.50
0.25
0.00
****
TRV2-GFP
TRV2-NbLecRK-IV.1

Supplementary Figure 2. Transient expression of StLecRK-IV.1-GFP, *NbLecRK-IV.1* VIGS construct and silencing efficiency. **A** StLecRK-IV.1-GFP and GFP were transiently expressed in *N. benthamiana* leaves 2 days after agroinfiltration. StLecRK-IV.1-GFP was detected by western blot using anti-GFP body. **B** Alignment of StLecRK-IV.1 and NbLecRK-IV.1 showing they share high similarity. **C** Structure of StLecRK-IV.1 and NbLecRK-IV.1. Position of the fragment used for *NbLecRK-IV.1* VIGS construction and for design RT-qPCR primers were indicated by line or arrow dash under NbLecRK-IV.1. The VIGS fragment specificity was detected on line (https://solgenomics.net). **D** Silencing of *NbLecRK-IV.1* did not affect the growth and development of *N. benthamiana* plant. **E** The bar graph showing silencing efficiency of *NbLecRK-IV.1* in *N. benthamiana*. The statistics were analysis by two-tailed t-test. Error bar represents ±SE.

Figure S3

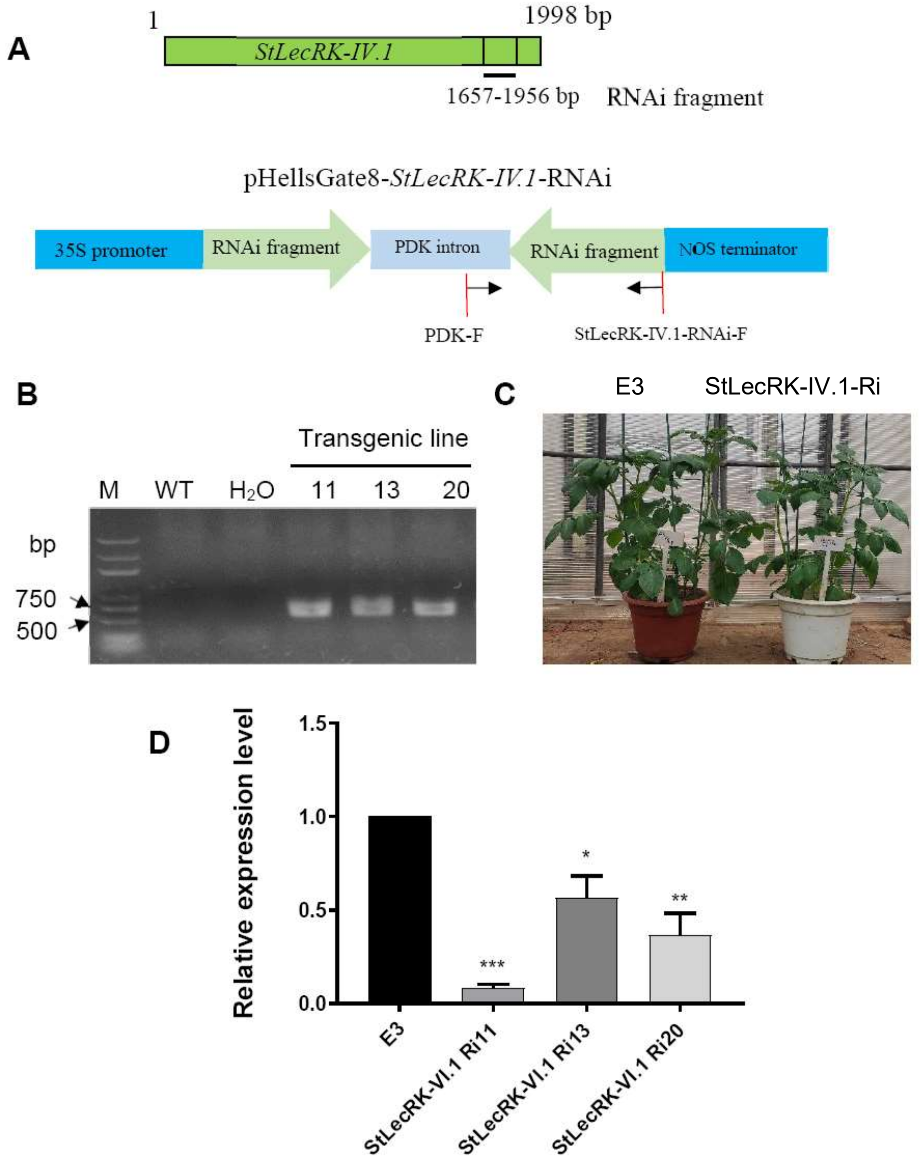

Supplementary Figure 3. *StLecRK-IV.1* interference construction and *StLecRK-IV.1* interference transgenic potato lines detection by RT-PCR. A Graphs showing the positions of the section used for interference vector construction and pHellsgate8-35S-StLecrk-IV.1-RNAi structure**. B** Gel image for testing the positive potato transgenic lines by genome PCR using vector primer PDK-F and gene specific primer StLecRK-IV.1-F. PCR run in 35 cycles. **C** The image showing that silencing of *StLecRK-IV.1* did not affect the growth of potato plants. **D** Silencing efficiency is shown by the mean fold change measured by qRT-PCR. E3 is wild type.

Figure S4

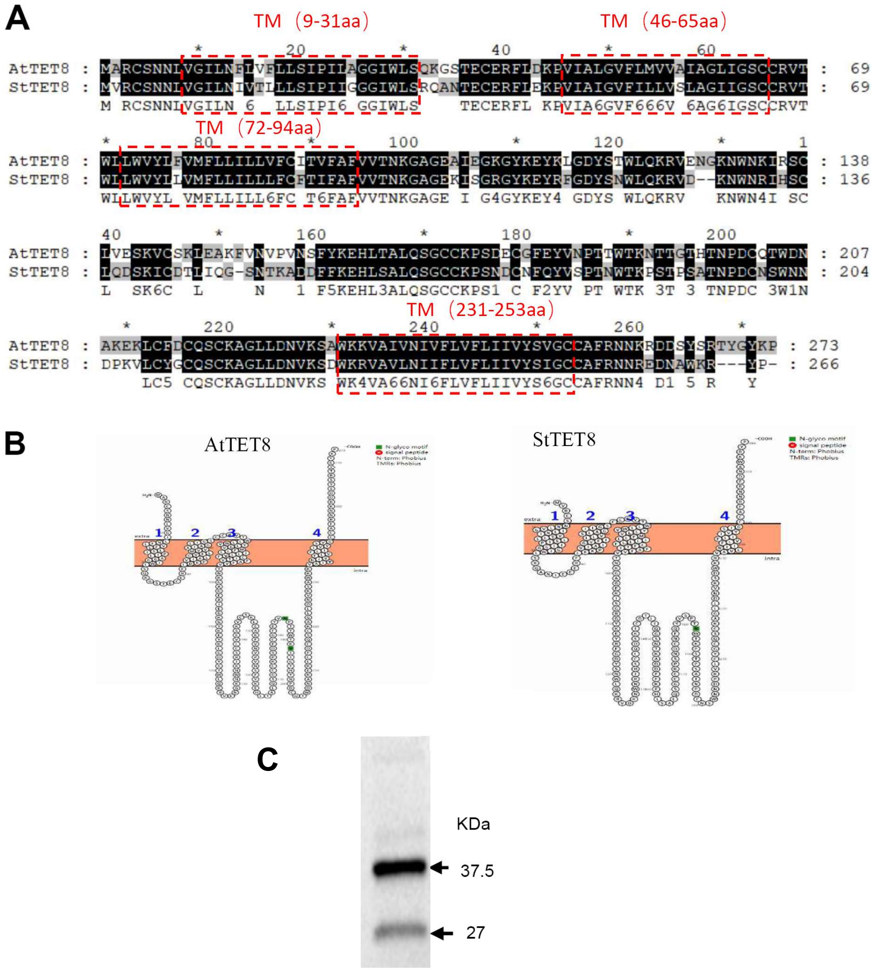

Supplementary Figure 4. Alignment of AtTET8 and StTET8 protein and their structure. A Alignment of TET8 from *Arabidopsis* (AtTET8, NP_850045.2) and TET8 from potato (StTET8, XP_006343564.1). **B** The predicted structures and the topology of StTET8 and AtTET8. Images were made using Protter (http://wlab.ethz.ch/protter/start/). **C** Western blot showing StTET8 was properly expressed in the functional verification assay in Fig. 4.

Figure S5

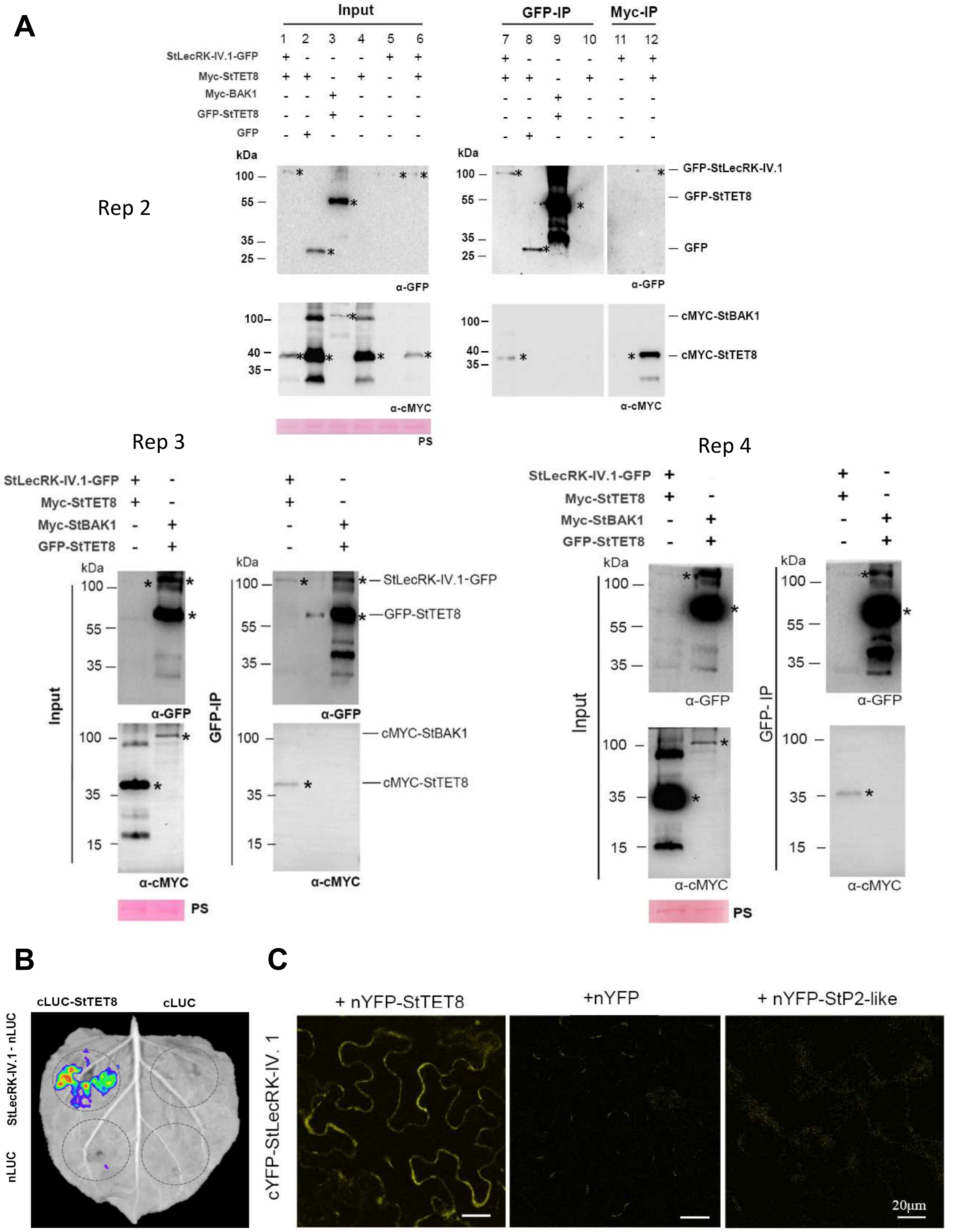

Supplementary Figure 5. Co-IP, luciferase complementation assay and BiFC confirm assotiation of StLecRK-IV.1 and StTET8. A Additional three repeats

showing StLecRK-IV.1 interacts with StTET8 by co-immunoprecipitation. Following pull-downs with GFP-trap beads, StLecRK-IV.1-GFP associated with cMyc-StTET8 (Lane 7) but EV-GFP-did not (Lane 8). And GFP-trap beads can not immunoprecipitate cMyc-StTET8 alone. GFP-StTET8 can not pull-down cMyc-BAK1. Following pull-downs with cMyc-trap beads, cMyc-StTET8 immunoprecipitated StLecRK-IV.1-GFP (lane 12) but cMyc-trap beads can not pull-down StLecRK-IV.1-GFP(Lane 11). Expression of constructs in *N. benthamiana* leaves is indicated by a + sign. Protein size markers are indicated in kilodaltons (kDa), and protein loading is indicated by Ponceau stain (PS). **B** luciferase complementation assay showing that StLecRK-IV.1 interacts with StTET8. **C** Image showing StTET8 and StLecRK-IV.1 interaction by BiFC assay. Images from left to right showing transient co-expression of cYFP-StLecRK-IV.1 with nYFP-StTET8, two negative controls: nYFP, and an un-interaction protein nYFP-StP2-like. OD_{600} of *Agrobacteria* suspension is 0.05 for split-YFP assay.

Figure S6

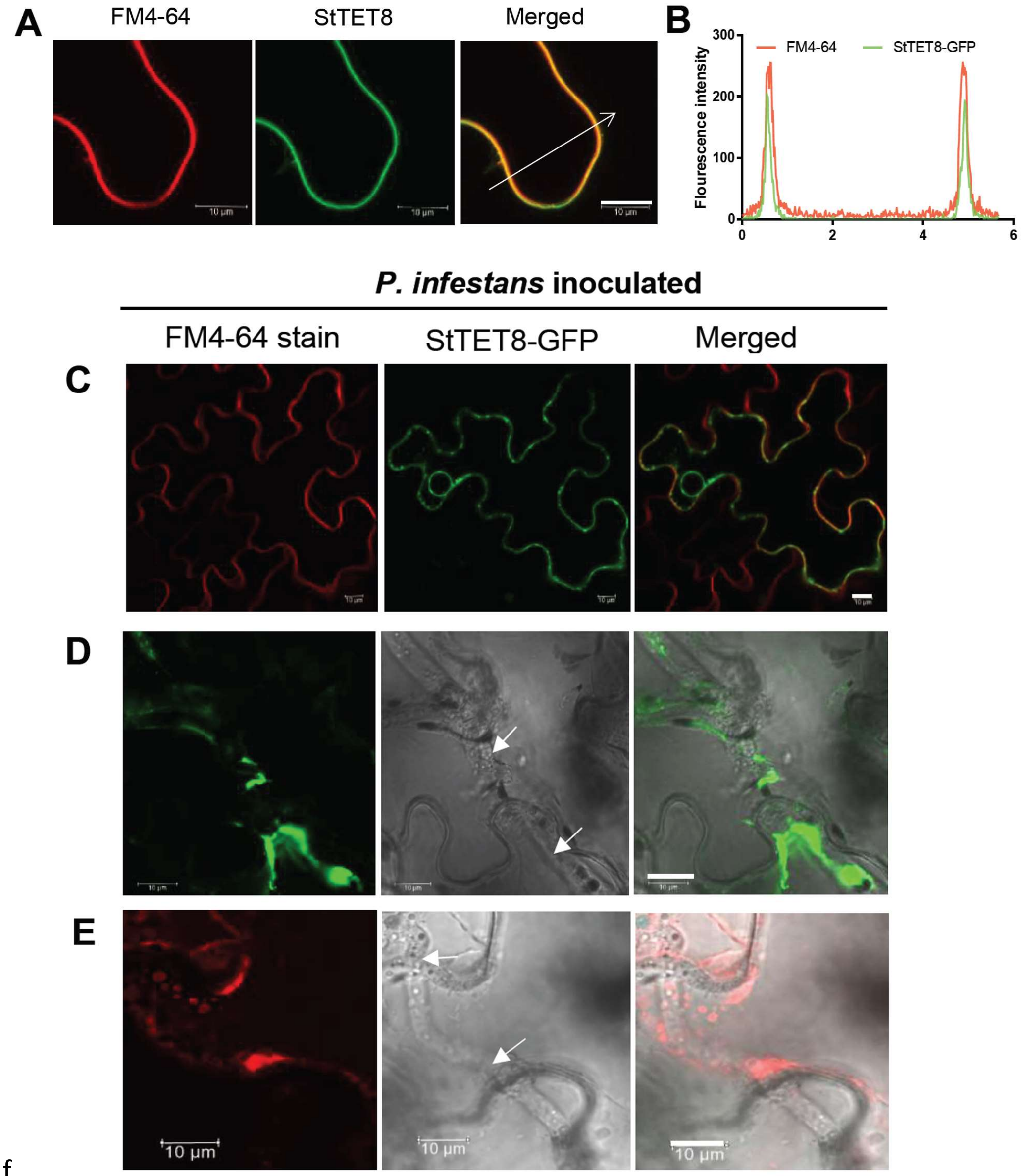

f

Supplementary Figure 6. Additional replicates (as in Figure 5) showing that StTET8 associated vesicles were triggered by *P. infestans* infection, and accumulated at the infection hyphae. A Images showing StTET8-GFP localization without *P. infestans* inoculation. **B** StTET8-GFP (green) and FM4-64 (red) fluorescence intensity plots across white arrows. **C** Compared with the water mock treatment in (A), more StTET8 associated vesicles was induced under the treatment with *P. infestans*. (**D-E**) Confocal images showing both StTET8-GFP (green fluorescence) and RFP-StTET8 (red fluorescence) associated vesicles accumulated around the hyphae of *P. infestans*. Arrows in D , E indicate the hyphae of *P. infestans*.

Figure S7

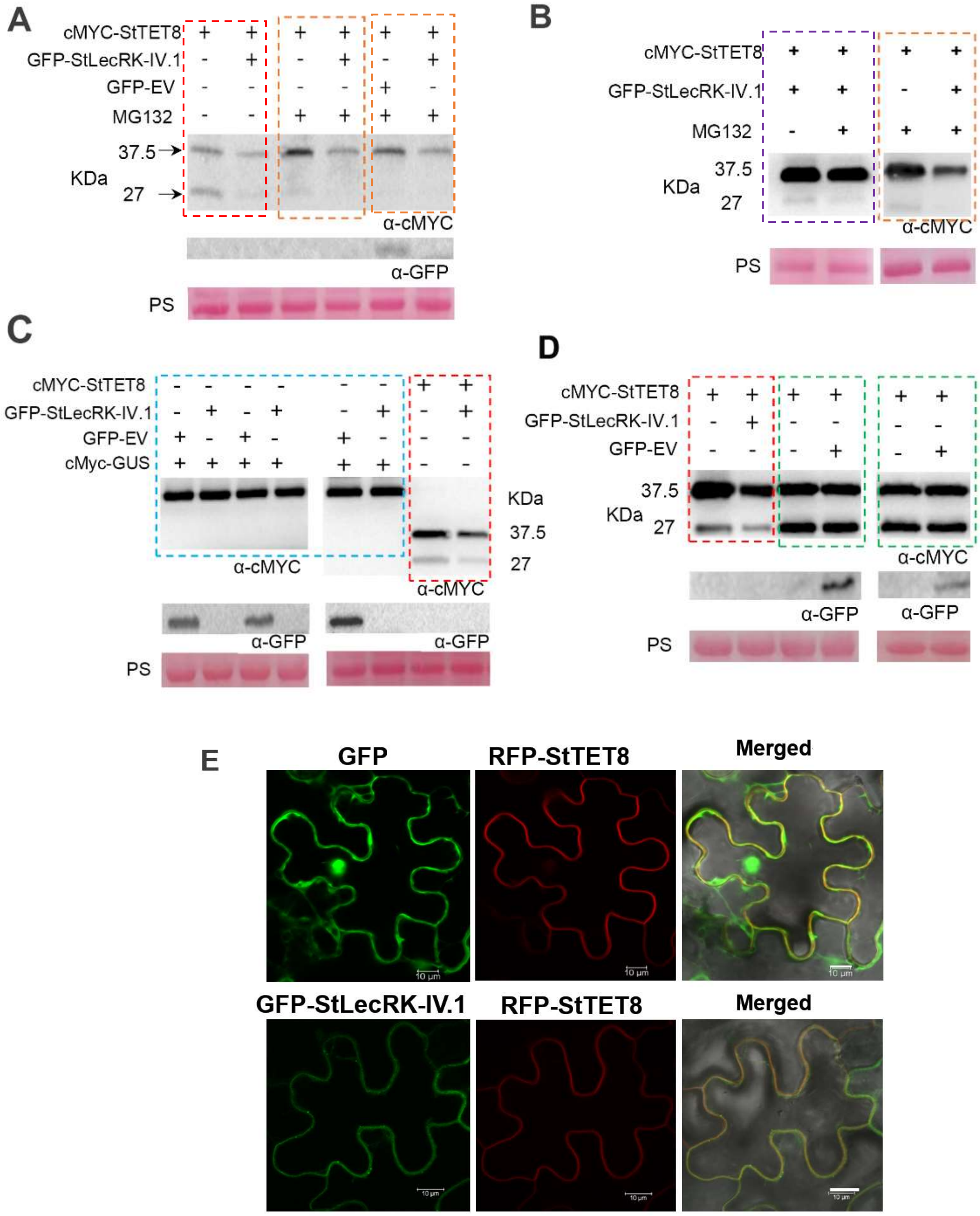

Supplementary Figure 7. Additional replicates (as in Figure 6) showing that the reduction of StTET8 when it was co-expressed with StLecRK-IV.1. Western blot indicates that StTET8 protein level was reduced significantly when it was co-expressed with StLecRK-IV.1 (shows in doted red box). Control cMyc-GUS protein did not reduce when it was co-expressed with StLecRK-IV.1 (shows in doted blue box) and the expression of GFP did not affect the StTET8 protein level (shows in green box). **A** and **B** The repeated assays indicate that StTET8 protein level was affected by

StLecRK-IV.1, and MG132 did not inhibit this affection (shows in doted orange box). **C** StLecRK-IV.1 did not induce cMyc-GUS reduction. **D** Western blot showing that co-expression of the control GFP with StTET8 did not affect the expression of StTET8. **E** Additional repeated assays of Fig. 6D showing expression of GFP-StLecRK-IV.1 leads to weak fluorescence intensity of RFP-StTET8 in same cell, versus it was co-expressed with GFP control. The samples were collected from the two side of the same leaf, and images were taken under the same parameters of confocal microscope. OD_{600} of *Agrobacteria* suspension is 0.1 for GFP and RFP constructs agroinfiltration.

Supplementary Table 1. Primers and constructs used in this study

Primer name	Sequencing（5'-3')	Use reference
attb1-StLecRK-IV.1-F	AAAAAAGCAGGCTTCATGGAGAAATTGTTTTTAGCCATG	C-terminal GFP fusion: StLecRK-IV.1-GFP<br>YFP StLecRK-IV.1-cYFP fusion
attb1-StLecRK-IV.1-R	CAAGAAAGCTGGGTTTCGACCACCGGAGAGAAG	
attb2-F	GGGGACAAGTTTGTACAAAAAAGCAGGCT	
attb2-R	GGGGACCACTTTGTACAAGAAAGCTGGGT	
qRT-StLecRK-IV.1-Q1-F:	CGATTTCACCTACAACGGGT	*StLecRK-IV.1* gene expression test and *StLecRK-IV.1-RNAi* transgenic potato silencing efficiency test
qRT-StLecRK-IV.1-Q1-R:	GACCCTCCTGCAATTGTGAA	
StLecRK-IV.1-RNAi-F	AAAAAAGCAGGCTTCGATCAAAGGGTATCGGACGAG	RNAi cloning primer for interference potato
StLecRK-IV.1-RNAi-R	CAAGAAAGCTGGGTTCCATGACATTGCAAAATGATCAAAG	
qRT-StEF1a-F	ATTGGAAACGGATATGCTCCA	Potato housekeeping gene
qRT-StEF1a-R	TCCTTACCTGAACGCCTGTCA	
NbLecRK-IV.1-VG-F	tgagtaaggttaccgaattcGGCTCAGTTGTTTCAACACAATA	VIGS cloning primer
NbLecRK-IV.1-VG-R	gtgagctcggtaccggatccCATGAGATACCCTCTTGACTGC	
TRV2-F	AAGGTTACCGAATTC	VIGS vector primer
TRV2-R	CTCGGTACCGGATCC	
NbLecRK-IV.1-Si-F	TGTGGGAGAAGGCCAATAGA	*NbLecRK-IV.1* silencing efficiency test
NbLecRK-IV.1-Si-R	TGGTCTAGCTGTTGGTTGTG	
NbActin-F	CAGAAAGGACCTCTACGGTAACAT	*N. benthamiana* housekeeping gene
NbActin-R	TCTGTGGACGATGGACGGAC	
StLecRK-IV.1-PBT3-SUC-F	ATTAACAAGGCCATTACGGCCATTTTTCAGTTTCTTGTTTTACTTTTTCATGCTCCATTAGGC	Y2H
StLecRK-IV.1-PBT3-SUC-R	AACTGATTGGCCGAGGCGGCCCCTCGACCACCGGAGAGAAGGGAATC	
PBT3-SUC-F	TGGCATGCATGTGCTCTG	Y2H
PBT3-SUC-R	GTAAGGTGGACTCCTTCT	
pPR3-N-F：	GTCGAAAATTCAAGACAAGG	Y2H
pPR3-N-R：	AAGCGTGACATAACTAATTAC	
attb1-StTET8-F	AAAAAAGCAGGCTTCATGGTGCGTTGTAGCAACAATTTAG	N-terminal RFP fusion: RFP-StLecRK-IV.1

attb1-StTET8-R	CAAGAAAGCTGGGTTTTAAGGATAACGCTTCCAAGCATTG	C-terminal GFP fusion: StTET8-GFP<br>YFP StTET8 -nYFP
Myc-StTET8-F	AttacgccgaggTCATGGTGCGTTGTAGCAACAATTTAG	3×myc
Myc-StTET8-R	TagggaagaggTCTTAAGGATAACGCTTCCAAGCATTG	
PDK-F	CTAATGCTAATATAACAAAGCGCAAGATC	pHellsgate8 vector primer
nLUC- StLecRK-IV.1-F	GACGAGCTCGGTACCATGGAGAAATTGTTTTTAGCCATGATTTTTC	nLUC fusion：nLUC- StLecRK-IV.1<br>cLUC fusion：cLUC-StTET8
nLUC-StLecRK-IV.1-R	CGAGATCTGGTCGACTCGACCACCGGAGAGAAG	
cLUC-StTET8-F	TCCCGGGGCGGTACCATGGTGCGTTGTAGCAACAATTTAG	
cLUC-StTET8-R	GCTCTGCAGGTCGACTTAAGGATAACGCTTCCAAGCATTG	
